# Transcriptomic and Metabolomic Investigation on Leaf Necrosis Induced by *ZmWus2* Transient Overexpression in *Nicotiana benthamiana*

**DOI:** 10.3390/ijms241311190

**Published:** 2023-07-07

**Authors:** Xianwen Zhang, Shuang Liang, Biao Luo, Zhongjing Zhou, Jiandong Bao, Ruiqiu Fang, Fang Wang, Xijiao Song, Zhenfeng Liao, Guang Chen, Yan Wang, Fei Xu, Yi Teng, Wanchang Li, Shengchun Xu, Fu-Cheng Lin

**Affiliations:** 1 Institute of Virology and Biotechnology, Zhejiang Academy of Agricultural Sciences, Hangzhou 310021, China; bestzxw@163.com (X.Z.); biaoluo@stu.hunau.edu.cn (B.L.); ffang0125@163.com (F.W.); wangy12429@163.com (Y.W.); li_wan_chang@163.com (W.L.); 2 State Key Laboratory for Managing Biotic and Chemical Threats to the Quality and Safety of Agro-Products, Zhejiang Academy of Agricultural Sciences, Hangzhou 310021, China; zj_20020101@163.com (Z.Z.); baojd@zaas.ac.cn (J.B.); songxijiao@zaas.ac.cn (X.S.); liaozhenfeng2@163.com (Z.L.); chenguang@zaas.ac.cn (G.C.); fxu_cl@outlook.com (F.X.); dgrty00@163.com (Y.T.); xusc@zaas.ac.cn (S.X.); 3 Hunan Engineering Laboratory for Good Agricultural Practice and Comprehensive Utilization of Famous-Region Medicinal Plants, Hunan Agricultural University, Changsha 410128, China; 4 Institute of Plant Protection and Microbiology, Zhejiang Academy of Agricultural Sciences, Hangzhou 310021, China; 5 Institute of Maize and Featured Upland Crops, Zhejiang Academy of Agricultural Sciences, Dongyang 322100, China; fangruiqiu2013@163.com; 6 Xianghu Laboratory, Hangzhou 311231, China

**Keywords:** *WUSCHEL*, necrosis, transcriptomics, metabolomics, tobacco, plant transgenic technology

## Abstract

*WUSCHEL* (WUS) is a crucial transcription factor in regulating plant stem cell development, and its expression can also improve genetic transformation. However, the ectopic expression of WUS always causes pleiotropic effects during genetic transformation, making it important to understand the regulatory mechanisms underlying these phenomena. In our study, we found that the transient expression of the maize WUS ortholog *ZmWus2* caused severe leaf necrosis in *Nicotiana benthamiana*. We performed transcriptomic and non-target metabolomic analyses on tobacco leaves during healthy to wilted states after *ZmWus2* transient overexpression. Transcriptomic analysis revealed that *ZmWus2* transformation caused active metabolism of inositol trisphosphate and glycerol-3-phosphate, while also upregulating plant hormone signaling and downregulating photosystem and protein folding pathways. Metabolomic analysis mainly identified changes in the synthesis of phenylpropanoid compounds and various lipid classes, including steroid synthesis. In addition, transcription factors such as ethylene-responsive factors (ERFs), the basic helix–loop–helix (bHLH) factors, and MYBs were found to be regulated by *ZmWus2*. By integrating these findings, we developed a WUS regulatory model that includes plant hormone accumulation, stress responses, lipid remodeling, and leaf necrosis. Our study sheds light on the mechanisms underlying WUS ectopic expression causing leaf necrosis and may inform the development of future genetic transformation strategies.

## 1. Introduction

*WUSCHEL* (WUS) is a homeodomain-containing transcription factor located in the organizing center of the shoot apical meristem (SAM) tissue in plants, where it plays a critical role in maintaining stem cell activity in the SAM [1,2,3]. WUS activates the negative regulator CLAVATA3 (CLV3) in cells adjacent to the central zone of the stem cell niche, creating a negative feedback loop that helps maintain the stem cell self-renewal and the apical dominance of the SAM [4,5]. There is a crosstalk between WUS and plant hormones, which are crucial for maintaining stem cell homeostasis in the shoot apex. On the one hand, WUS controls auxin levels and enhances cytokinin output to prevent stem cells from differentiation in response to auxin. On the other hand, the proper gradient level of auxin in the specific SAM region is also a prerequisite for the induction of WUS activity [6,7,8].

Transgenic technology has been widely used to improve plant phenotypic traits, and somatic embryogenesis (SE) plays a critical role in plant genetic transformation. The traditional approach involves introducing modified DNA into cultured cells, triggering callus formation and organogenesis by exposure to high concentrations of plant hormones. However, this process is time-consuming, inefficient, and highly genotype-dependent [9]. Over the past two decades, studies have shown that ectopic expression of *AtWus* in *Arabidopsis thaliana* promotes the vegetative-to-embryonic transition [10]. Furthermore, the morphogenic transcription factors (MTFs) *ZmBbm* and *ZmWus2* can bypass the callus culture stage and directly stimulate SE, greatly improving transformation efficiency [11,12]. In addition, ectopic overexpression of WUS alone can also promote transformation efficiency in plants [13]. Consequently, ectopic expression of MTFs has been widely used in various crops, such as maize, sorghum, tobacco, sugarcane, and rice [12,14,15,16]. However, the constitutive expression of MTFs can result in serious pleiotropic effects. For example, persistent expression of *ZmBbm* and *ZmWus2* in the monocot maize resulted in abnormally thick roots, stunted growth, and sterility in T0 transgenic plants [15]. Additionally, the T1 seeds containing the Nospro: *Wus2* expression box showed inconsistent germination [14]. In the case of tobacco plants transfected by *ZmWus2*, developmental abnormalities like curled leaves were observed [16]. Although some methods have been proposed to address these issues, including excision of the WUS gene after SE, or limiting the expression of the MTFs at specific developmental stages [11,17], little is known about the mechanisms behind the pleiotropic effects caused by WUS, which limits our ability to fully utilize this technology.

To investigate the molecular mechanism of plant pleiotropic effects caused by WUS overexpression, we transiently expressed *ZmWus2* in leaves of the model plant tobacco (*Nicotiana benthamiana*) and observed changes in plant morphology. As expected, tobacco leaves exhibited a wilting phenotype at 48 h post-infiltration (hpi) and were completely necrotic at 96 hpi. To further understand the underlying reasons of WUS-triggered leaf necrosis, we dynamically monitored molecular changes within tobacco leaves at 24 hpi, 48 hpi, and 66 hpi after WUS transfection using transcriptomics and non-target metabolomics. By integrating the omics results, we ultimately deduced that WUS mainly induced significant alterations in four aspects: hormone changes, stress responses, lipid remodeling, and necrosis-related processes in the leaves. These findings elucidate how WUS promotes SE and instigates plant pleiotropic effects. Moreover, our study sheds light on the molecular mechanisms underlying leaf wilt caused by WUS transfection in tobacco, offering valuable insights for the improvement of highly efficient genetic transformation strategies.

## 2. Results

### 2.1. Transient Expression of ZmWus2 Leads to Tobacco Leaf Necrosis

**Figure 1:**
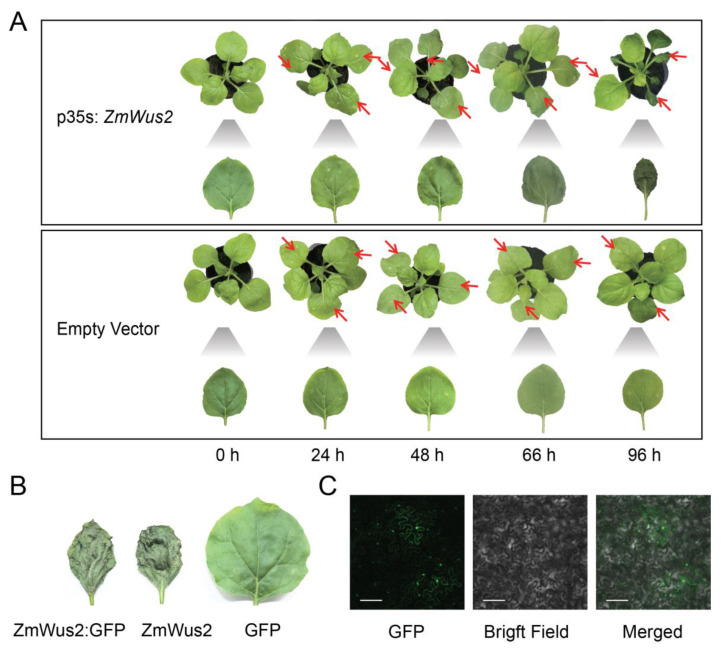
Leaf necrosis caused by *ZmWus2* transfection. (**A**) The phenotypes of tobacco leaves after *ZmWus2* transfection. The location of the injection is marked by the red arrow. By magnifying the structure of the leaves, dehydration began to appear in the leaves injected with p35s: *ZmWus2* at 48 hpi and the leaves completely wilted by 96 hpi. (**B**) The morphology of tobacco leaves transfected by p35s: *ZmWus2*: *GFP*, p35s: *ZmWus2*, and p35s: GFP, respectively, for 96 h. Both *ZmWus2*: *GFP* and *ZmWus2* vectors caused the leaves to wilt. (**C**) The GFP fluorescence observation of leaves transfected by p35s: *ZmWus2*: *GFP* at 48 hpi. Scale Bar = 120 μm.

Tobacco leaves were transfected with p35s: *ZmWus2* vector (Figure S1) for transient expression by agroinfiltration, and an empty vector was used as a control. At 24 hpi, the plant leaf showed no visible changes. However, at 48 hpi, the tobacco leaves infiltrated with the *ZmWus2* began to show wilting symptoms, and by 96 hpi, they were completely wilted (Figure 1A). In contrast, the tobacco leaves transfected with the empty vector showed no obvious changes and maintained a healthy phenotype (Figure 1A). Using transmission electron microscopy (TEM), we observed that tobacco leaves infiltrated with the *ZmWus2* gene showed signs of necrosis at 66 hpi, including organelle swelling, cell membrane rupture, and degradation of the cytoplasm and nucleus (Figure S2). These are evident indicators of leaf necrosis. In contrast, the organelle structure in control leaves remained normal (Figure S2).

To observe the expression and localization of the *ZmWus2* in leaves, we specifically infiltrated tobacco leaves with p35s: *ZmWus2*: *GFP* (Figure S1). The tobacco leaves exhibited necrosis at 66 hpi, consistent with the patterns observed in leaves treated with p35s: *ZmWus2*, while those treated with p35s: *GFP* remained healthy (Figure 1B). Fluorescence observation confirmed the expression of *ZmWus2* in leaves at 48 hpi, with most fluorescence detected in the nucleus (Figure 1C).

### 2.2. Transcriptome Changes in Tobacco Leaves Induced by ZmWus2 Transfection

**Figure 2:**
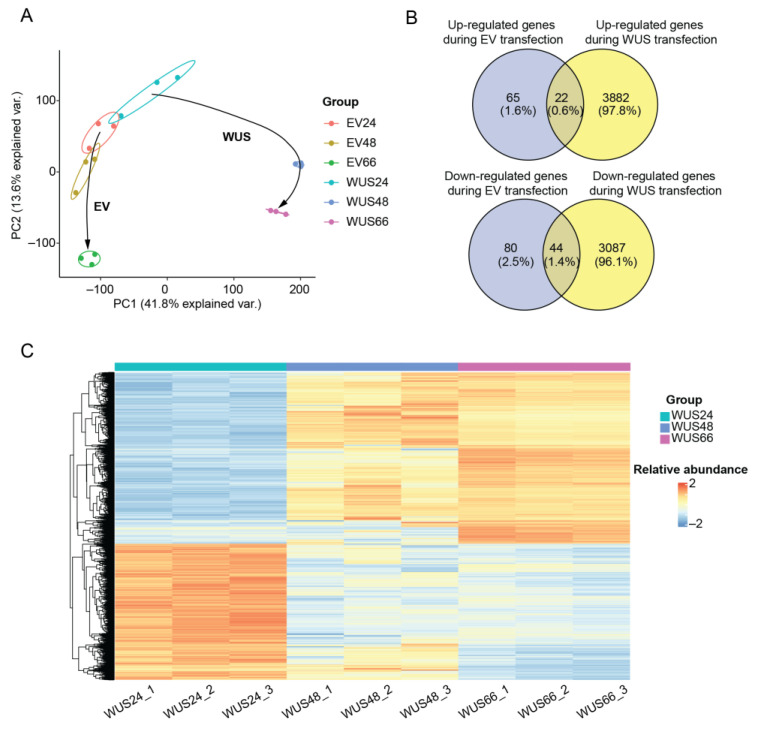
Transcriptomic analysis of tobacco leaves transfected by WUS. (**A**) PCA plot of the transcriptome. The trajectory of changes for the WUS and EV groups are labeled with arrows. (**B**) Venn diagram analysis of DEGs. Dysregulated genes that are only presented in the WUS group are considered WUS-induced DEGs. (**C**) Heatmap overview of all 6969 DEGs. The genes were scaled across all samples, where blue indicates relatively low expression and red indicates relatively high expression in the respective sample. The DEGs underwent unsupervised clustering using complete linkage and Euclidean distance.

To investigate the regulatory role of *WUSCHEL *on gene expression, transcriptomic analysis was performed on tobacco leaves induced by *ZmWus2* transfection collected at 24, 48, and 66 hpi (WUS24, WUS48, and WUS66 groups), as well as a control set of plant leaves that were transfected with an empty vector at the same time points (EV24, EV48, and EV66) (Table S1). A total of 45,493 genes were characterized, with an average of 42,401 ± 467 genes identified in each group (Figure S3A). The transcriptome was clustered by the group under unsupervised clustering PCA analysis. At 24 hpi, the WUS24 group was clustered closely to the EV24 group, indicating that *ZmWus2* had not yet significantly affected gene expression at this time point. The WUS 48 group showed the greatest distance in both the first and second principal components compared to the WUS24 group, and the distance between WUS48 and WUS66 was mainly reflected in the second principal component. The transcriptome of the EV group showed only time changes in the second principal component (Figure 2A). We then analyzed the differential expressed genes (DEGs) between WUS24 and WUS66 groups. A total of 3904 DEGs were identified to be upregulated and 3131 DEGs were downregulated (Figure S3B). To confirm that the DEGs were specifically induced by WUS transfection, we screened genes whose expression was affected by EV transfection and used them as background genes to extract DEGs regulated by WUS (Figure S3C). Finally, a total of 6969 DEGs were identified, including 3882 upregulated and 3087 downregulated genes (Figure 2B,C).

### 2.3. Biological Insights through Transcriptomic DEGs

**Figure 3:**
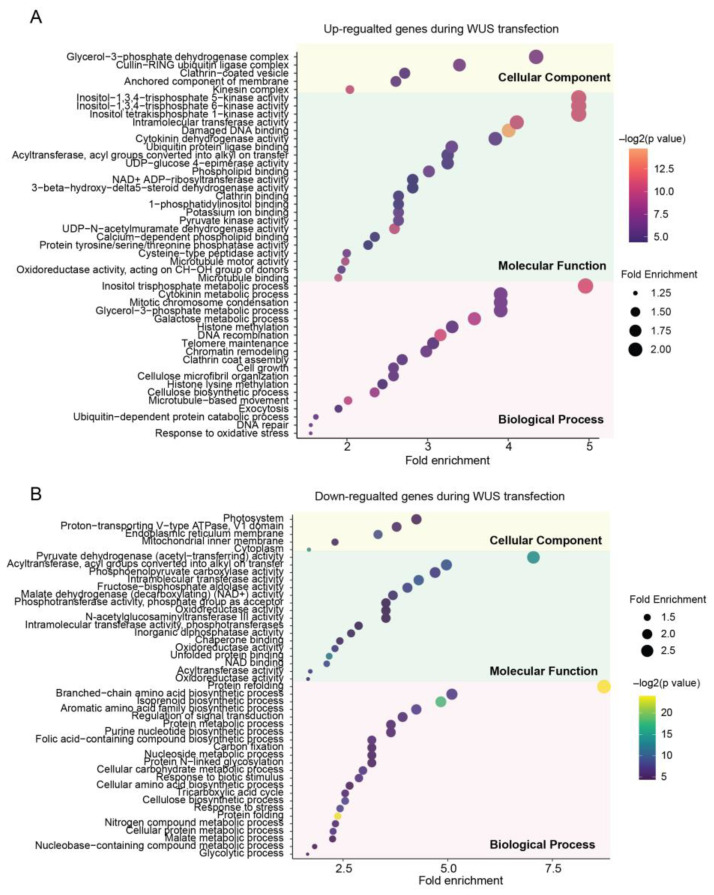
GO enrichment analyses of upregulated DEGs (**A**) and downregulated DEGs (**B**), respectively. All significantly enriched entries are listed, where the horizontal axis and dot size represent the enrichment fold change of the respective entry.

To gain a better understanding of the biological significance of the DEGs, Gene Ontology (GO) enrichment was performed (Table S2). The most enriched biology processes (BPs) of the upregulated DEGs were the inositol trisphosphate metabolic process, cytokinin metabolic process, mitotic chromosome condensation, and glycerol-3-phosphate metabolic process, while the downregulated DEGs were mainly involved in protein refolding (Figure 3A,B). In terms of molecular function (MF), the upregulated DEGs were primarily related to inositol trisphosphate kinase activity, intramolecular transferase activity, as well as damage DNA binding, and the top enriched MFs of downregulated DEGs were pyruvate dehydrogenase activity, acyltransferase, phosphoenolpyruvate carboxylase activity. (Figure 3A,B). Regarding the cellular component (CC), the glycerol-3-phosphate dehydrogenase complex was the most enriched in upregulated DEGs, while the photosystem was the most enriched in downregulated DEGs.

The inositol trisphosphate plays an important role in plant responses to abiotic stresses. As part of the response to a stimulus, inositol trisphosphate levels increase transiently [18,19]. The upregulated DEGs enriched in the GO term of damaged DNA binding also indicate that plant cells are undergoing stresses [20]. Glycerol-3-phosphate, which was also reported related to plant defense against stress, was upregulated during the redifferentiation phase of SE of coffee [21]. The cytokinin metabolic process and mitotic chromosome condensation were important processes of cell division, which were also found to be upregulated in the transcriptomes during SE of *Arabidopsis* and *Coffea arabica* [22,23]. The downregulation of DEGs related to the photosystem indicates weakened photosynthesis in the leaves, consistent with the visible phenotype of leaf wilting, while the downregulated protein refolding process may indicate the presence of abnormal protein folding processes, which is a key feature of leaf necrosis. In summary, transcriptomic data revealed that *ZmWus2* transfection caused stress in tobacco leaves and maintained a high level of cell division. At the same time, protein folding and photosynthesis in the leaf were damaged.

### 2.4. Metabolomic Changes in Tobacco Leaves Induced by ZmWus2 Transfection

**Figure 4:**
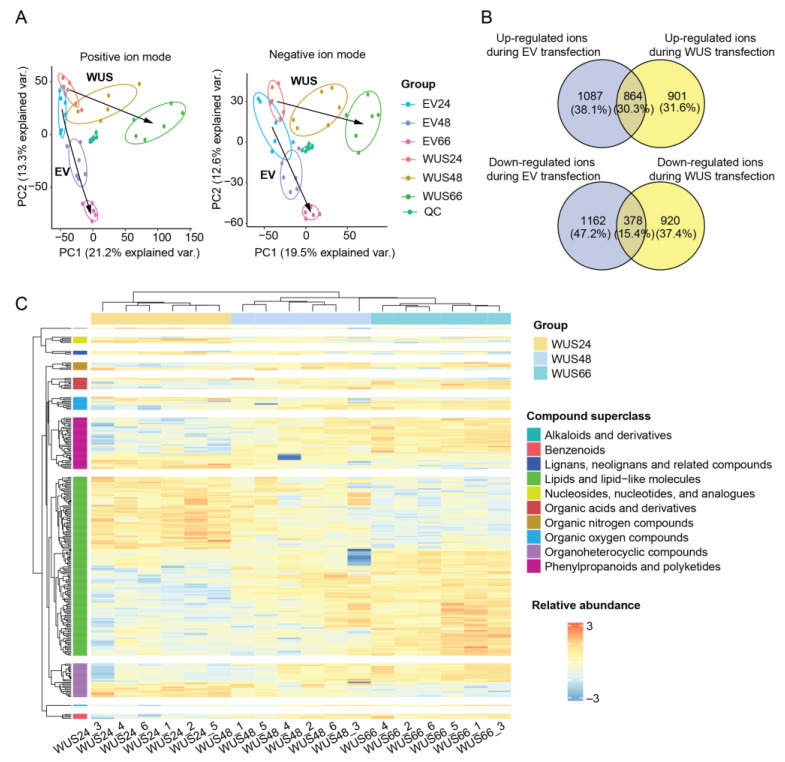
Metabolomic analysis of tobacco leaves transfected by *ZmWus2*. (**A**) PCA plot of the metabolome. The trajectory of changes for the WUS and EV groups are labeled with arrows. (**B**) Venn diagram analysis of differential ions. Dysregulated ions that are only presented in the WUS group are considered WUS-induced differential ions. (**C**) Heatmap overview of all 240 DEMs. The metabolites were scaled across all samples, where blue indicates relatively low expression and red indicates relatively high expression in the respective sample. The DEMs of each compound superclass underwent unsupervised clustering using complete linkage and Euclidean distance.

We also investigated the regulated metabolome profiles induced by *ZmWus2* (Table S3). Non-targeted metabolomics revealed a total of 17,354 ions, including 10,642 positive ions as well as 6712 negative ions, with an average of 17,001 ± 52 ions per group (Figure S4A). PCA clustering analysis revealed that all samples were segregated into groups, indicating that the experimental conditions had a significant impact on the metabolomic profiles. Furthermore, the quality control (QC) samples, which were pooled from all the individual samples, were tightly clustered in the center of the plot, indicating the high data quality (Figure 3A). The WUS24 and EV24 groups were highly similar, indicating that little change occurred in the metabolic profiles of tobacco leaves at 24 hpi after *ZmWus2* transfection. However, unlike the transcriptomic data, which showed the most drastic changes between 24 hpi and 48 hpi, the metabolomic data exhibited sustained and dramatic changes over the time course of 24–48–66 hpi, as evidenced by the strong loading of the first principal component in the PCA plot (Figure 4A). Transfection with the EV caused changes in both the first and second principal components of leaf metabolome (Figure 4A). Through comparing the metabolome of WUS24 and WUS66, we identified 1651 ion features that were upregulated and 1325 that were downregulated (Figure S4B). After subtracting the background metabolic changes caused by the EV transfection (Figure S4C), we confirmed a total of 1725, including 786 upregulated and 939 downregulated differentially expressed ions (Figure 4B).

### 2.5. Functional Annotation of Metabolomic DEMs

**Figure 5:**
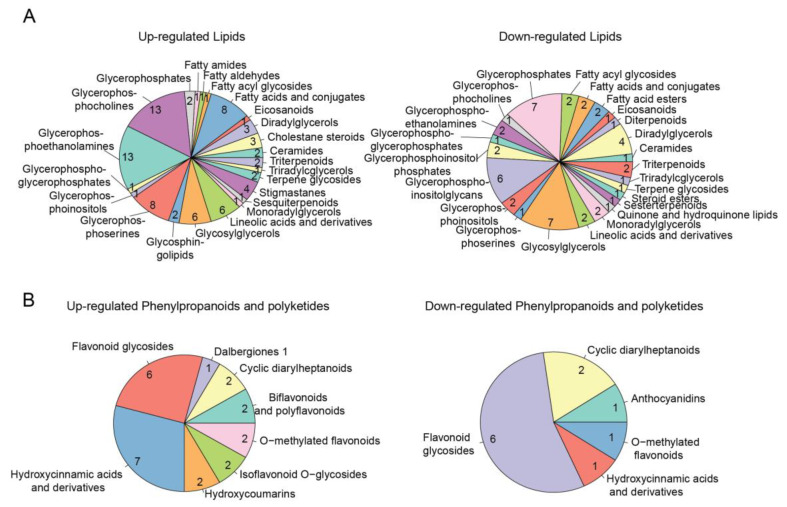
Pie charts were used to display the main classes of differentially upregulated or downregulated lipids (**A**) and phenylpropanoids (**B**), respectively.

After comparing to compound databases, we annotated 240 differential expressed metabolites (DEMs) out of the 1725 ions. Of these DEMs, 150 were upregulated while 90 were downregulated (Figure 4C, Table S4). In terms of chemical classification, both the upregulated DEMs and the downregulated DEMs mainly belonged to the superclass of lipids and lipid-like molecules, as well as phenylpropanoids and polyketides (Figure S4D,E). The most upregulated lipids were glycerophosphocholines (GPC) and glycerophosphoethanolamines (GPE), while the most downregulated lipids were glycerophosphates (GP) and glycosylglycerols (GG) (Figure 5A). These lipid structures are important components of the membrane. Their changes indicate the remodeling of the membranes inside the leaf cells. Lipid remodeling, especially of the phospholipids in the membrane, has been proven to be an essential adaptation mechanism for plants to cope with stresses [24]. The most upregulated phenylpropanoids and polyketides were hydroxycinnamic acids and flavonoid glycosides, and the most downregulated phenylpropanoids were flavonoid glycosides (Figure 5B). Phenylpropanoids are important components of the plant cell wall. They have antioxidant and free radical scavenging properties and play a role in coping with biotic or abiotic stress, including infection, injury, exposure to pollutants, and other adverse environmental conditions [25]. Indeed, previous studies have reported that the SE process of avocado leads to the accumulation of phenylpropanoids, which are believed to increase cell wall thickness and enhance stress tolerance. Furthermore, cinnamic acid and ferulic acid were found to stimulate SE production in avocado [26]. Therefore, the changes in phenylpropanoids and polyketides caused by *ZmWus2* transfection may be linked to both stress response and embryonic development.

### 2.6. Metabolic Pathways Regulated by ZmWus2

**Figure 6:**
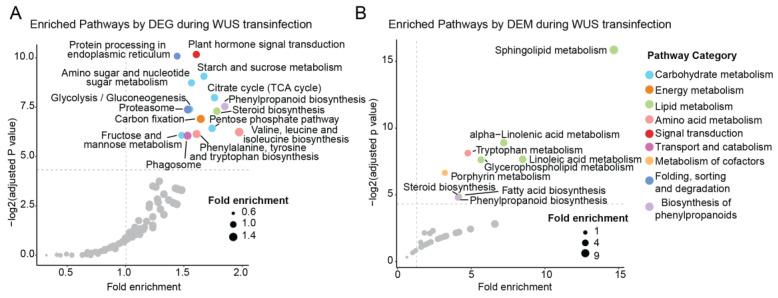
The KEGG enrichment analyses of DEGs (**A**) and DEMs (**B**). Pathways with BH-adjusted *p*-values < 0.05 and fold enrichment >1 were considered significantly enriched, and their names were labeled in the figure. The metabolic category of each pathway was distinguished by color.

By integrating transcriptomic and metabolomic data, we were able to identify genes and compounds involved in metabolic pathways that were regulated by WUS. We performed KEGG pathway enrichment analyses on the DEGs and DEMs separately and found that a total of 14 transcriptomic pathways were significantly enriched, including plant hormone signal transduction, carbohydrate metabolism, amino acid metabolism, phenylpropanoid biosynthesis, lipid metabolism, energy metabolism, and folding, sorting, and degradation (Figure 6A, Table S5). Notably, the transcriptomic data highlighted the disturbance of carbohydrate metabolism, with six enriched pathways: starch and sucrose metabolism, amino sugar and nucleotide sugar metabolism, glycolysis, TCA cycle, pentose phosphate pathway, as well as fructose and mannose metabolism (Figure 6A). As an energy source, carbohydrates have been shown to positively affect both the number of somatic embryos and the increase in callus fresh weight, which was found to be over-accumulated during the dedifferentiation, callose, redifferentiation, and embryo stages [27]. On the other hand, nine metabolic pathways were significantly enriched, including pathways belonging to lipid metabolism, amino acid metabolism, phenylpropanoid biosynthesis, and cofactor metabolism (Figure 6B, Table S6). The metabolomic data in this study emphasized the disruption of lipid metabolism, with five enriched pathways consisting of sphingolipid, alpha-linolenic acid, linoleic acid, and glycerophospholipid metabolism, as well as steroid biosynthesis (Figure 6B). As discussed above, lipid remodeling caused by WUS transfection may reveal stress within its leaves. Additionally, the pathway of porphyrin metabolism with six compounds, including chlorophyll B, chlorophyll derivatives, and their precursors were enriched (Figure S5A). Five of the six metabolites were downregulated during WUS transfection, which is consistent with the finding of a downregulated photosystem in the transcriptome.

### 2.7. Regulated Metabolic Pathways Evident in Both Transcriptome and Metabolome

**Figure 7:**
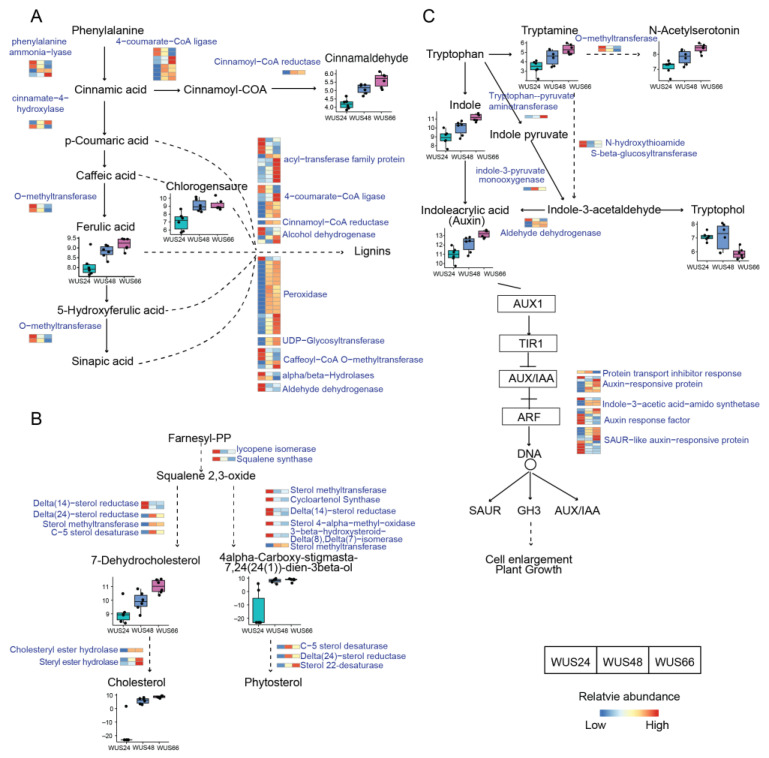
Regulated metabolic pathways. (**A**) The phenylpropanoids biosynthesis pathway, (**B**) the steroid biosynthesis pathway, and (**C**) tryptophan metabolism coupled with its downstream auxin signaling pathway. These pathways were simplified based on the KEGG pathways, where solid lines represented direct reactions and dashed lines represented indirect reactions with multiple steps. The metabolites were shown in black font, while enzymes were depicted in blue font. DEMs associated with this pathway were presented using boxplots, showing their abundance changes following WUS transfection. The *y*-axis of the boxplot represents the log2-transformed relative abundance of the compounds. DEGs associated with this pathway were represented using a heatmap, showing their expression changes following WUS transfection. Blue indicated low expression, while red indicated high expression.

Both transcriptome and metabolome data indicated the enrichment of three pathways: phenylpropanoid biosynthesis, steroid biosynthesis, and tryptophan metabolism coupled with its downstream auxin signaling pathway. The phenylpropanoid biosynthesis pathway involved the transformation of phenylalanine to cinnamic acid, which finally turned into lignins. During WUS transfection, ferulic acid, caffeoylquinic acid, and cinnamaldehyde were detected to accumulate (Figure 7A), and 12 enzymes were enriched, most of which were upregulated. Additionally, 38 phenylpropanoids were detected in DEMs that were not matched into this pathway (Figure S5B). For steroid biosynthesis, 7-dehydrocholesterol, cholesterol, and intermediate products of phytosterol were found to be elevated (Figure 7B). Finally, both omics confirmed the enrichment of tryptophan metabolism, and the accumulation of indoleacrylic acid (one of the auxin modes) was also detected in the metabolome. The integration of the two pathways confirmed the auxin biosynthesis from tryptophan and the enhanced auxin signaling (Figure 7C). Auxin is considered one of the most important factors inducing SE, and the accumulation of auxin after WUS transfection in leaves may partly explain the increased efficiency of SE induced by WUS. Additionally, the transcriptomic data revealed DEGs participating in other plant hormone signaling pathways, including cytokinin, abscisic acid (ABA), ethylene, brassinosteroid (BR), and jasmonic acid (JA) (Figure S6).

### 2.8. Transcription Factor Regulated by ZmWus2

**Figure 8:**
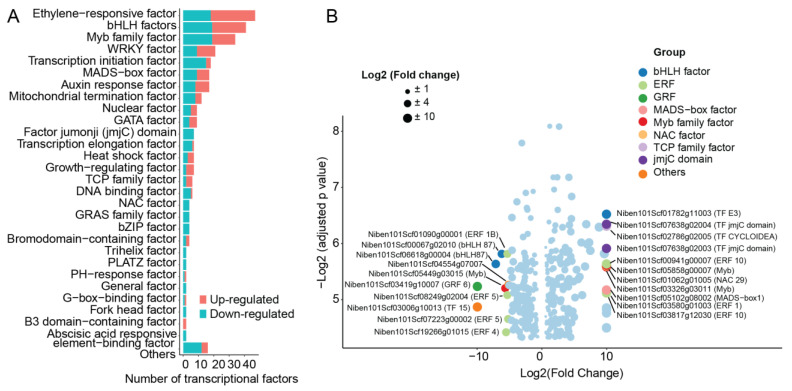
Dysregulated TFs after WUS transfection. (**A**) Numbers of each category of dysregulated TF. The red bars indicated upregulated, while the green bars indicated downregulated. (**B**) The actual change in differentially expressed TFs. The *x*-axis indicated the log2 transformed fold change value of the TFs, and the *y*-axis represented significance. The most upregulated and downregulated TFs were labeled in the figure, along with their categories.

As a transcription factor (TF), WUS has been reported to widely affect the expression of its downstream TFs [28]. In our data, among the 6969 DEGs, a total of 313 transcription factors were found to be dysregulated during WUS transfection. The largest proportion of dysregulated TFs were the 47 ethylene response factors (ERFs), followed by 41 the basic helix–loop–helix (bHLH) factors, 34 MYB factors, and 21 WRKY factors (Figure 8A). Among all transcription factors, the bHLH E3, TF of the factor jumonji (jmjC) domain showed the most significant upregulation with the highest fold change and significant statistical difference. TFs MYB, NAC29, MADS-box1, ERF10, and ERF1 also exhibited substantial upregulation. The most significantly downregulated transcription factor was ERF1B, followed by bHLH 87, MYB, growth regulating factor 6 (GFR6), TF 15, ERF5, ERF4, etc. (Figure 8B). This finding is consistent with the changes observed in TFs during SE induction in *Arabidopsis*, specifically involving ERF, WRKY, MYB, and bHLH [29,30]. The TFs bHLH, ERF, MYB, and WRKY were all widely reported to regulate the processes of plant growth and development, environmental stresses, and secondary metabolite synthesis [31,32,33]. In particular, TF belonging to bHLH as well as ERF has been proven to impact the induction of SE in *Arabidopsis* [34,35]. Additionally, we also identified transcription factors that are involved in the regulation of plant hormone signaling, including auxin response factors (ARFs) and abscisic acid response element-binding factors (Figure 8A). This emphasizes the role of WUS in regulating plant hormone signaling.

**Figure 9:**
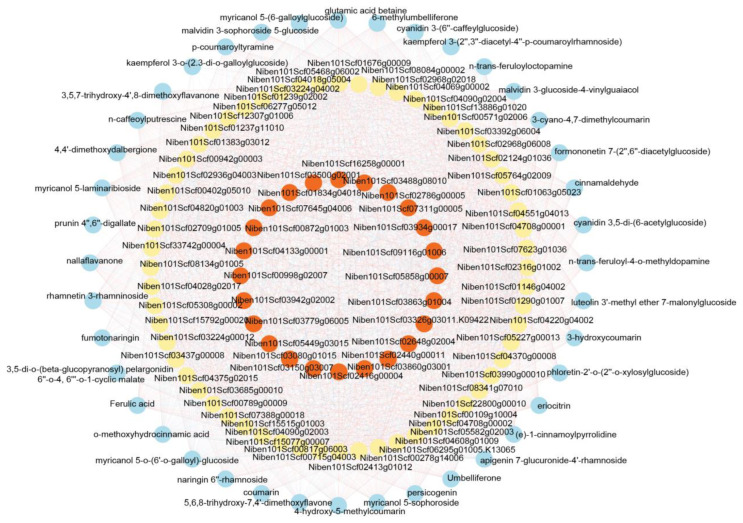
The co-expression network of MYB TFs regulating phenylpropanoid metabolism. The red circles were MYB TFs, the yellow circles represented enzymes in phenylpropanoid biosynthesis, and the blue circles were phenylpropanoids. Their regulation connections were represented with edges, where red indicated a positive correlation and blue indicated a negative correlation.

MYB TFs have been extensively reported to regulate the synthesis and metabolism of phenylpropanoids [36,37]. Therefore, we investigated the regulatory network of MYB TFs about phenylpropanoid metabolism. Among the 25 differentially expressed MYB TFs, 24 were found to be strongly correlated with 60 enzymes involved in phenylpropanoid metabolism. A total of 525 correlations were established, including 252 positive and 273 negative correlations (Figure 9, Table S7). These regulated enzymes further catalyze the metabolism of various compounds and establish 1974 correlations with phenylpropanoids, including 1170 positive and 804 negative correlations (Figure 9, Table S7). These results revealed that upstream WUS regulates downstream MYB TFs, which further affect the metabolism of phenylpropanoids.

## 3. Discussion

**Figure 10:**
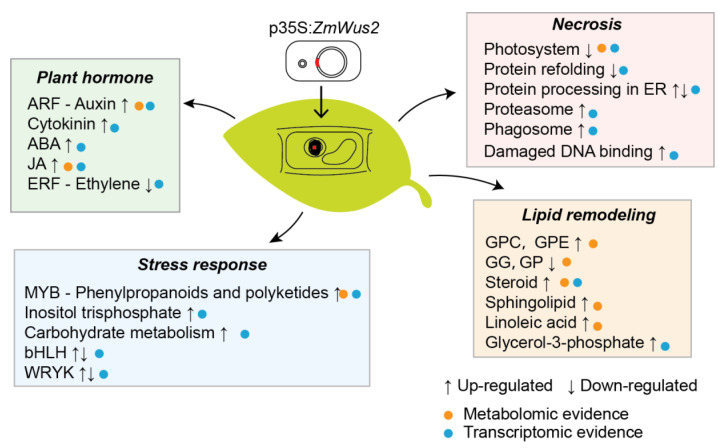
The molecular changes caused by WUS transfection in tobacco leaves, summarized from four aspects: hormone changes, stress response, lipid remodeling, and necrosis. Blue circles represented information obtained from transcriptomics, while orange circles represented messages obtained from metabolomics. The symbol “↑” indicates up-regulated processes caused by WUS transfection, while “↓” indicates down-regulated processes.

*WUSCHEL* is the essential factor for de novo establishment of the shoot stem cell niche, and it also plays a pivotal role in inducing the direct SE in tissue culture. However, constitutive expression of WUS can cause serious pleiotropic effects. To investigate the negative effects of ectopic WUS expression in plants, we transfected *ZmWus2* into tobacco leaves. The results revealed that overexpression of WUS caused leaf dehydration at 48 hpi and complete withering at 96 hpi, with TEM-confirmed leaf necrosis accompanied by organelle rupture. Although previous studies have reported that overexpression of WUS can directly induce SE, our study did not observe the formation of SE due to the complete withering of tobacco leaves within four days and a lack of proper tissue culture conditions. Nevertheless, through transcriptomic and metabolomic analyses during WUS transfection, we observed abundant molecular changes consistent with molecular changes reported that occurred in the early SE. Here, we mainly discussed these molecular changes from four perspectives (Figure 10).

Firstly, the overexpression of WUS induced dysregulated signaling pathways of multiple plant hormones, with the most significant being the accumulation of auxin (Figure 7C). Previous studies have shown that WUS inhibits auxin levels in the SAM and promotes abscisic acid signaling through the negative feedback loop with CLV to maintain the activity of stem cell division [7]. However, in this study, WUS was ectopically expressed in tobacco leaves, and the loss of CLV regulation may cause changes in its function. The induction of SE requires a large amount of auxin. In practical production, the exogenous addition of growth hormone analog 2,4-D is often used to induce the dedifferentiation of tissues. Endogenous auxin levels are also important for SE. A gradient of auxin needs to be established in specific regions to form somatic embryos [38]. In the early SE of coffee, high levels of endogenous auxin have been observed [39]. Consistent with our results, the expression of genes involved in auxin synthesis was upregulated in another MTF *Bbm*-induced SE [40]. However, while auxin is necessary for early SE and callus formation, it is not essential for embryo maturation in plants. In the presence of continuous hormone stimulation, carrot cell lines can develop into the globular embryo stage but cannot further develop [41].

In addition to changes in auxin levels, transcriptome data also showed alterations in other hormone-signaling pathways. We observed the upregulation of cytokinin metabolism (Figure 3A) and dysregulated downstream genes involved in cytokinin signaling (Figure S6). Some reports suggested that cytokinin can also induce SE [42]. In addition, the downstream genes involved in abscisic acid signaling were upregulated, while most genes involved in ethylene signaling were downregulated (Figure S6). In addition, abscisic acid and ethylene have also been reported to participate in SE [43,44]. Moreover, an upregulation of the jasmonic acid pathway was observed (Figure S6), which is involved in defense against biotic and abiotic stresses. A-linolenic acid is the precursor of JA synthesis, and the metabolic pathway of a-linolenic acid was found to be upregulated in the metabolome (Figure 6B). Therefore, although the content of JA was not directly detected, we have confirmed the accumulation of JA and its signal transduction pathway upregulation after WUS transfection. Recent research has proved that JA can stimulate auxin biosynthesis and induce SE [45]. The transcriptome data also identified genes involved in brassinosteroid and salicylic acid signaling pathways (Figure S6). Overall, these findings suggest that overexpression of WUS leads to dramatic changes in the levels of plant hormones and their signaling pathways, which may be one of the important reasons for promoting early SE.

Secondly, besides alterations in plant hormones, the majority of molecular changes induced by WUS have been reported to be associated with stress tolerance. One of the most typical changes is the accumulation of phenylpropanoids and alterations in their regulatory TFs MYB. Phenylpropanoids are a large class of plant secondary metabolites including flavonoids, lignins, phenolic acids, stilbenes, and coumarins. They play an important role in cell wall formation and have been reported to function under environmental stress. In *Nicotiana*, the accumulation of phenylpropanoids has been associated with nitrogen and boron deficiencies, as well as pathogen infection [46,47,48].

In addition to phenylpropanoids and MYB, other molecules such as inositol trisphosphate, carbohydrates, plant hormone JA, TF bHLH, and TF WRYK have also been reported to be involved in plant stress resistance. Previous studies have reported that WUS is associated with stress resistance. WUS has been shown to prevent cucumber mosaic virus (CMV) from accumulating in the central and peripheral areas of meristematic tissues. The accumulation of these stress-related molecules also indirectly indicates that WUS transfection causes a state of internal stress in the plant [17,49]. Interestingly, in addition to plant growth regulators like 2,4-D, some external stresses have been reported to promote SE [50]. Therefore, we suggest that WUS overexpression may induce internal stress in plants, and the plants themselves trigger stress response processes by expressing compounds such as phenylpropanoids.

Thirdly, the results of metabolomics and transcriptomics analyses also discovered the lipid remodeling induced by WUS overexpression, with content changes of kinds of lipids including sterols, sphingolipids, linoleic acid, glycerol-3-phosphate, GPC, GPE, GP, and GG. The role of lipid remodeling in WUS overexpression or SE induction is not clear, with only limited evidence suggesting that lipid remodeling does occur during SE development and may be related to stress resistance [51]. It is worth noticing that we observed the upregulation of intermediate products of phytosterol in the steroid pathway in our metabolomic data, which is likely to promote the production of plant steroids. Brassinosteroid is one of the essential plant steroid hormones, whose dysregulated signaling pathway has been detected in our transcriptomic data (Figure S6). The BR signaling has been reported to be modulated by SE co-receptor kinases [52]. Therefore, the increase in sterols may be regulated by changes in SE-related signaling molecules induced by WUS overexpression.

Lastly, aside from the aforementioned molecular alterations, WUS transfection in tobacco leaves resulted in severe wilting phenotype, and TEM revealed internal organelle damage, nuclear dissolution, and serious necrosis. In *Nicotiana benthamiana*, overexpression of recombinant proteins can sometimes lead to the accumulation of unfolded or misfolded proteins, causing leaf necrosis and dehydration [53]. In our experiments, the overexpression of *ZmWus2* through the p35s promoter caused leaf necrosis in tobacco, while overexpression of GFP through the same p35s promoter showed a healthy phenotype, which may indicate that *ZmWus2* does cause a molecular mechanism of leaf necrosis. We have found that there existed internal stress after WUS transfection, and it has been reported that when plants are subjected to abiotic stress, plant cells undergo hypersensitivity reactions and quickly initiate cell death to protect the host from further stress damage [54]. Therefore, stress induced by WUS may be one of the reasons for leaf necrosis. In addition, various molecular changes related to necrosis were found in our transcriptomic data, including downregulated photosystem, downregulation of protein folding, damaged DNA binding, dysregulated protein processing in the endoplasmic reticulum, dysfunctional protein degradation pathways including proteasome and phagosome (Figure 6A).

In summary, this study systematically analyzed the transcriptome and metabolome of tobacco leaves during WUS transfection, and summarized the internal molecular changes caused by WUS in the aspects of hormones, stress, lipids, and necrosis (Figure 10).

## 4. Materials and Methods

### 4.1. Construction of a Binary Vector for Tobacco Transformation

The *ZmWus2* and *GFP* genes were synthesized to match the nucleotide sequences of *ZmWus2* (GenBank: NM_001112491.1) and *EGFP* (GenBank: MN517551), respectively. The binary vector was created by substituting the hygromycin-resistant gene of the pCambia 1300 plasmid (predigested with XhoI) with *ZmWus2*, *EGFP*, or *ZmWus2*: *EGFP*, resulting in the generation of vectors named 1300-WUS, 1300-GFP, and 1300-WUS-GFP. The fusion gene *ZmWus2*: *EGFP* was linked with a linker peptide containing the coding sequence “ggaggaggaggtggtagc”. Additionally, the hygromycin-resistant gene was deleted from the 1300 plasmid through XhoI digestion to generate an empty vector.

### 4.2. Agroinfiltration of Tobacco Leaves

One day prior to agroinfiltration, tobacco plantlets were incubated at 20 °C with dim light. The agroinfiltration process involved using the second, third, and fourth leaves of 4-week-old tobacco plantlets, excluding the youngest leaf due to difficulties in infiltration. To prepare for infiltration, bacterial cells were harvested by centrifugation and then suspended in a solution containing 10 mM MES (pH 5.6), 10 mM MgCl2, and 150 μM acetosyringone. The suspension was adjusted to a final concentration that corresponds to an absorbance of 1.5 at 600 nm and incubated at 28 °C for 3 h. Next, the bacterial cell suspension was injected into the entire leaf area through a scratch using a 1 ml plastic syringe without a needle. Following the infiltration, the plantlets were placed in a growth chamber with controlled conditions.

### 4.3. Subcellular Localization

At 48 hpi, a leaf tissue measuring 1 × 1 cm was collected from the region closest to the syringe wound. The collected leaf tissues expressing p35s: *ZmWus2*: *GFP* were subjected to confocal imaging using a Leica TCS SP2 confocal imaging system. GFP fluorescence was captured using an excitation wavelength of 488 nm and emission detection in the range of 500–530 nm. For each localization experiment, a minimum of three independent samples per plant were visualized, with each sample corresponding to a single infiltrated leaf.

### 4.4. Ultra-Thin Section and Semi-Thin Section

The tobacco leaves were initially fixed in a solution of 2.5% glutaraldehyde in 0.1 M phosphate buffer with a pH of 7.0 for a duration of 12 h. After fixation, they were rinsed with phosphate buffer and further treated with 1% osmium tetroxide for 2 h, followed by another rinse with phosphate buffer. Next, the samples underwent dehydration using a series of ethanol solutions and were then embedded in Spurr’s epoxy. Thin-microtome was used to prepare tissue sections with a thickness of 1–2 μm and ultrathin sections with a thickness of 70–90 nm. The tissue sections were mounted and stained with 1% methylene blue, while the ultrathin sections were successively mounted and stained with 1% uranyl acetate and 1% lead citrate. Finally, the tissue sections and ultrathin sections were photographed using a VHX-2000 microscope (Keyence, Osaka, Japan) and a H-7650 Transmission Electron Microscope (Hitachi, Ibaraki, Japan), respectively.

### 4.5. Transcriptomic Analysis

For RNA sequencing, the plant leaves were subjected to total RNA extraction using Trizol Reagent. The quantity of RNA was determined using a NanoDrop spectrophotometer. mRNA isolation was performed using poly-T oligo-attached magnetic beads, followed by fragmentation using divalent cations in an Illumina proprietary buffer. First-strand cDNA synthesis utilized random oligonucleotides and Super Script II, while second-strand cDNA synthesis involved DNA Polymerase I and RNase H. Blunt ends were generated by exonuclease/polymerase activities, and adapters were ligated after adenylation of the DNA fragments’ 3’ ends. The Illumina PCR primer cocktail enriched DNA fragments with adapters via a 15-cycle PCR reaction. Purification of 400–500 bp PCR products was achieved using the AMPure XP system, and quantification was performed with the Agilent Bioanalyzer 2100 system. Library construction for sequencing was conducted on the NovaSeq 6000 platform (Illumina, San Diego, CA, USA). Cutadapt (v1.15) software was employed to filter the sequencing data, generating high-quality clean data for subsequent analysis. In silico read mapping was performed using HISAT2 v2.0.5 with the Nicotiana benthamiana draft genome sequence v1.0.1 and gene annotation files from the Sol Genomics Network as the reference genome. HTSeq (v0.9.1) statistics were utilized for comparing Read Count values, which served as the initial gene expression.

### 4.6. Metabolomic Analysis

For metabolomic preparation, freeze-dried tobacco leaf powder was extracted by 70% methanol through sonication on ice for 60 min. For mass spectrometry analysis, a reversed-phase ACQUITY UPLC BEH C18 column (2.1 × 100 mm, 1.7 µm particle size, Waters) coupled to an ACQUITY I-Class UHPLC separation system and a VION IMS QTOF mass spectrometer equipped with an electrospray ionization (ESI) interface (Waters) was used. Gradient elution was performed using mobile phase A (98% H2O, 2% ACN, 0.01% FA) and mobile phase B (98% ACN, 2% H2O, 0.01% FA). The eluent composition of mobile phase B was initially set at 5% for 0.5 min. It was then increased linearly over 25.5 min to reach 100%. This composition was maintained for 2.5 min before returning to the initial conditions for 1.5 min. The column temperature was set at 40 °C, and the flow rate was 0.4 mL/min. Mass spectra were acquired from *m*/*z* 50 to 1000. The positive and negative ESI modes were maintained at 3 kV and 2.5 kV, respectively. Lock mass correction was achieved by continuously infusing a leucine-enkephalin solution every 1 min during data acquisition. Samples were acquired using the HDMS^E^ mode. Progenesis QI Informatics (Nonlinear Dynamics, Waters, Milford, MA, USA) was employed for data processing and compound identification. Commercial databases Metlin, NIST, and plant metabolite databases like KNApSAcK [55] and CMAUP [56] were utilized for metabolite identification. Compounds with a total score greater than 30 and a fragmentation score greater than 20 were considered confidently annotated (Level 2).

### 4.7. Data Statistics

The transcriptomic read counts were normalized using DESeq2 and log2 transformed along with the metabolomic data to achieve normal distribution. Genes or ions with missing values below 90% were retained for further analysis, with missing values replaced by minimum values. Differential expression analysis was performed on genes or ions exhibiting a fold change value greater than 1.5 or less than 0.67, as well as a Benjamini and Hochberg adjusted Welch’s *t*-test *p*-value below 0.05 between comparison groups, identifying them as differentially expressed (DEG or DEM). The Gene Ontology (GO) and KEGG Orthology (KO) annotations for DEGs were obtained from Sol Genomics. Classification and KO annotations for DEMs were conducted using ClassyFire [57] (https://cfb.fiehnlab.ucdavis.edu/, accessed on 1 March 2023) and MetaboAnalyst [58] (https://www.metaboanalyst.ca/MetaboAnalyst, accessed on 20 March 2023), respectively. GO and pathway enrichment analyses were performed using the enricher function in the R package clusterProfiler 4.6.0. Significant enrichment was determined based on a *p*-value below 0.05 and a fold enrichment value (GeneRatio/BgRatio) above 1.5 for GO enrichment, and a *p*-value below 0.05 and a fold enrichment above 1 for KEGG enrichment. Connections with Pearson correlations greater than 0.8 and *p*-values less than 0.05 between transcription factors and enzymes, as well as between enzymes and related metabolites, were considered upstream or downstream regulatory connections. Visualization of the network was achieved using Cytoscape.

## 5. Conclusions

In this study, through transcriptomic and non-targeted metabolomic analyses, we studied the regulation patterns of gene expression and metabolite contents during leaf wilting caused by *ZmWus2* transfection, as well as downstream transcription factors regulated by WUS. Integrating the results of differential transcriptome and metabolome, we developed a regulatory model of WUS transfection that included plant hormone changes characterized by auxin accumulation, stress response represented by phenylpropanoid biosynthesis and MYB transcription factors, lipid remodeling represented by steroid biosynthesis, and molecular changes associated with leaf necrosis. As WUS is widely used to promote plant transformation efficiency, we believe that a detailed understanding of the internal molecular changes caused by WUS will provide further biological insights for better use of plant transformation.

## Data Availability

The study contains the original findings, which are provided in the article and Supplementary Materials. For additional inquiries, please contact the corresponding author S.L.

## Supplementary Materials

The supporting information can be downloaded at: https://www.mdpi.com/article/10.3390/ijms241311190/s1.
