# Supplementary material for: Transcriptomic and Metabolomic Investigation on Leaf Necrosis Induced by ZmWus2 Transient Overexpression in Nicotiana benthamiana
Source: Int J Mol Sci. 2023 Jul 7;24(13):11190. doi: 10.3390/ijms241311190 (PMC10342293; doi:10.3390/ijms241311190)
Supplement: Supplementary file 1 [file ijms-24-11190-s001.zip › ijms-2476729-supplementary-Figure.pdf]

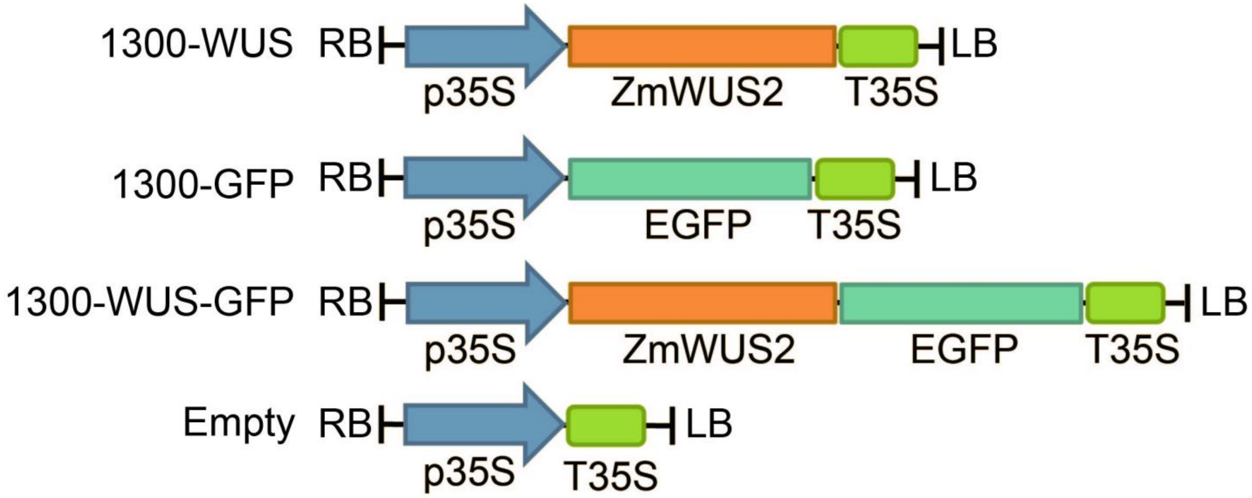

Figure S1. Vectors used in this study.1300-WUS indicate ZmWUS2 gene overexpressing vector,1300-GFP indicate EGFP gene overexpressing vector, 1300-WUS-GFP indicate ZmWUS2-EGFP fusion gene overexpressing vector, Empty indicate blank control vector. p35S indicate cauliflower mosaic virus 35S promoter, T35S indicate cauliflower mosaic virus 35S terminator, ZmWUS2 indicate WUS2 gene in maize, EGFP indicate enhanced green fluorescent protein gene. RB indicate the right boundary, LB indicate the left boundary.

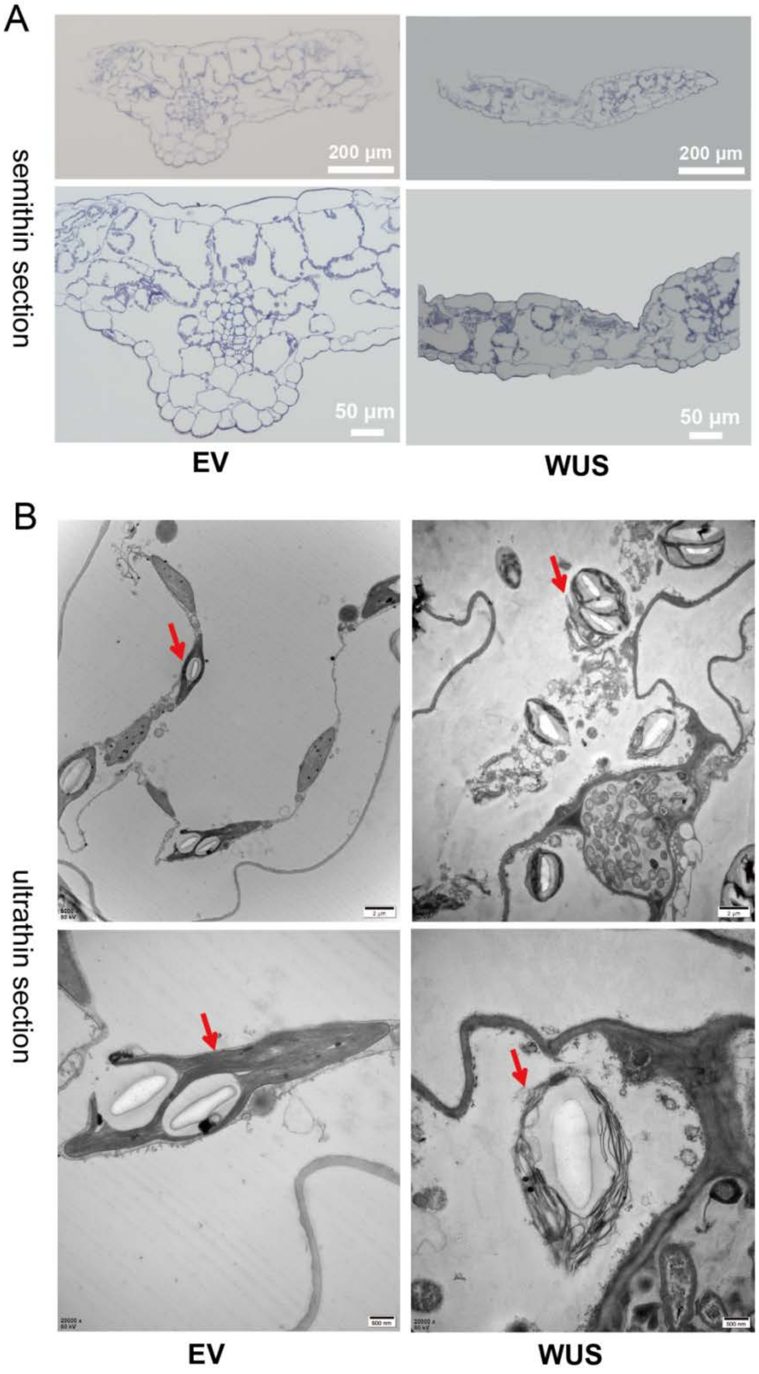

Figure S2. *ZmWUS2* gene transient expression cause collapse of leaf cell structure and deformation of organelle of *Nicotiana benthamiana*. (**A**) semithin section, (**B**) ultrathin section. The red arrow in (B) represents the chloroplast.

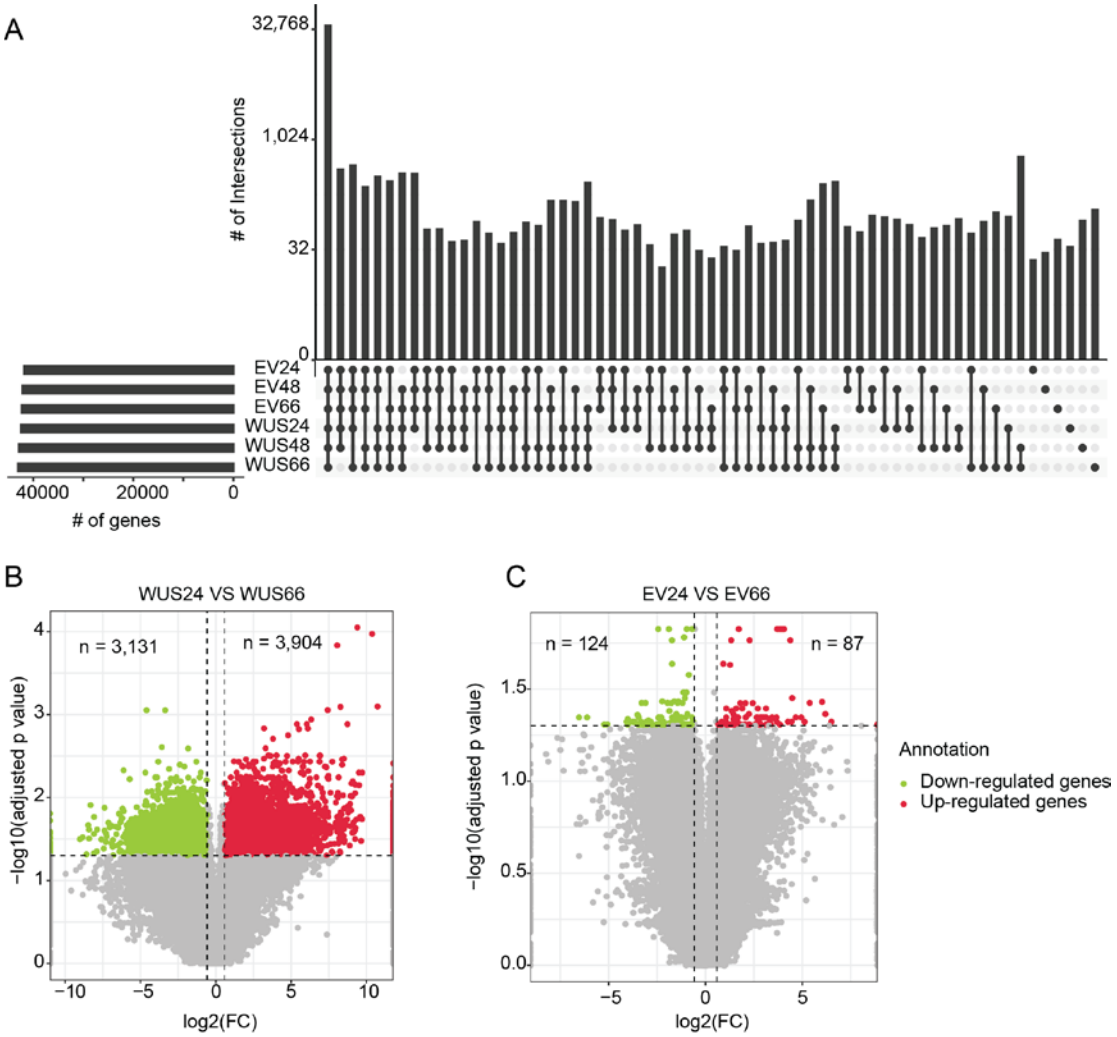

Figure S3. (**A**) Upset plot represented the quantified gene numbers in each group. (**B**) The volcano plot showed the up-regulated (red points) as well as down-regulated genes (green points) in the WUS66 group compared to WUS24. (**C**) The volcano plot showed the up-regulated (red points) as well as down-regulated genes (green points) in the EV66 group compared to EV24.

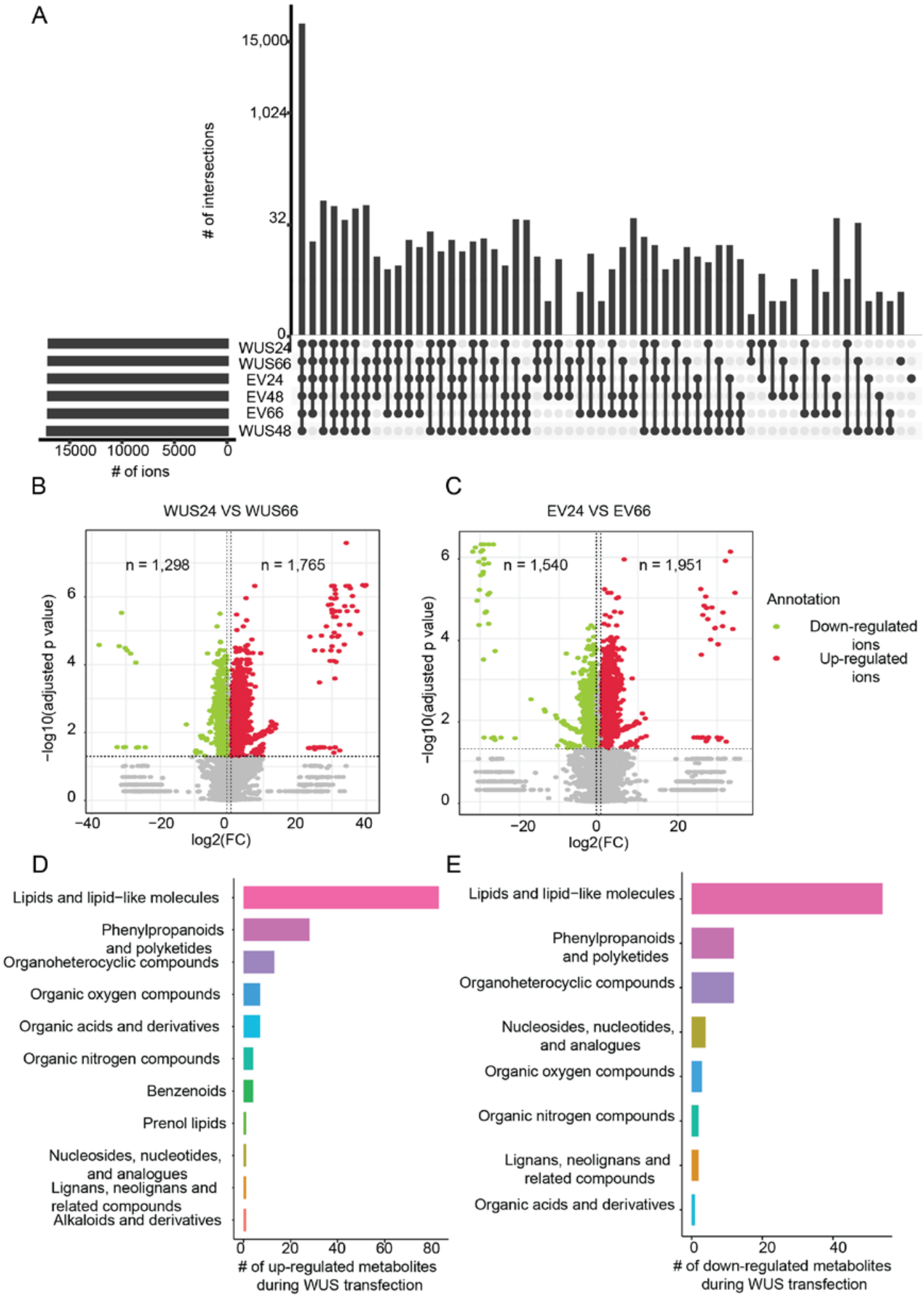

Figure S4. (**A**) Upset plot represented the quantified gene numbers in each group. (**B**) The volcano plot showed the up-regulated (red points) as well as down-regulated ions (green points) in the WUS66 group

compared to WUS24. (**C**) The volcano plot showed the up-regulated (red points) as well as down-regulated ions (green points) the in EV66 group compared to EV24. (**D**) The structure superclasses of up-regulated DEMs. (E) The structure superclasses of down-regulated DEMs.

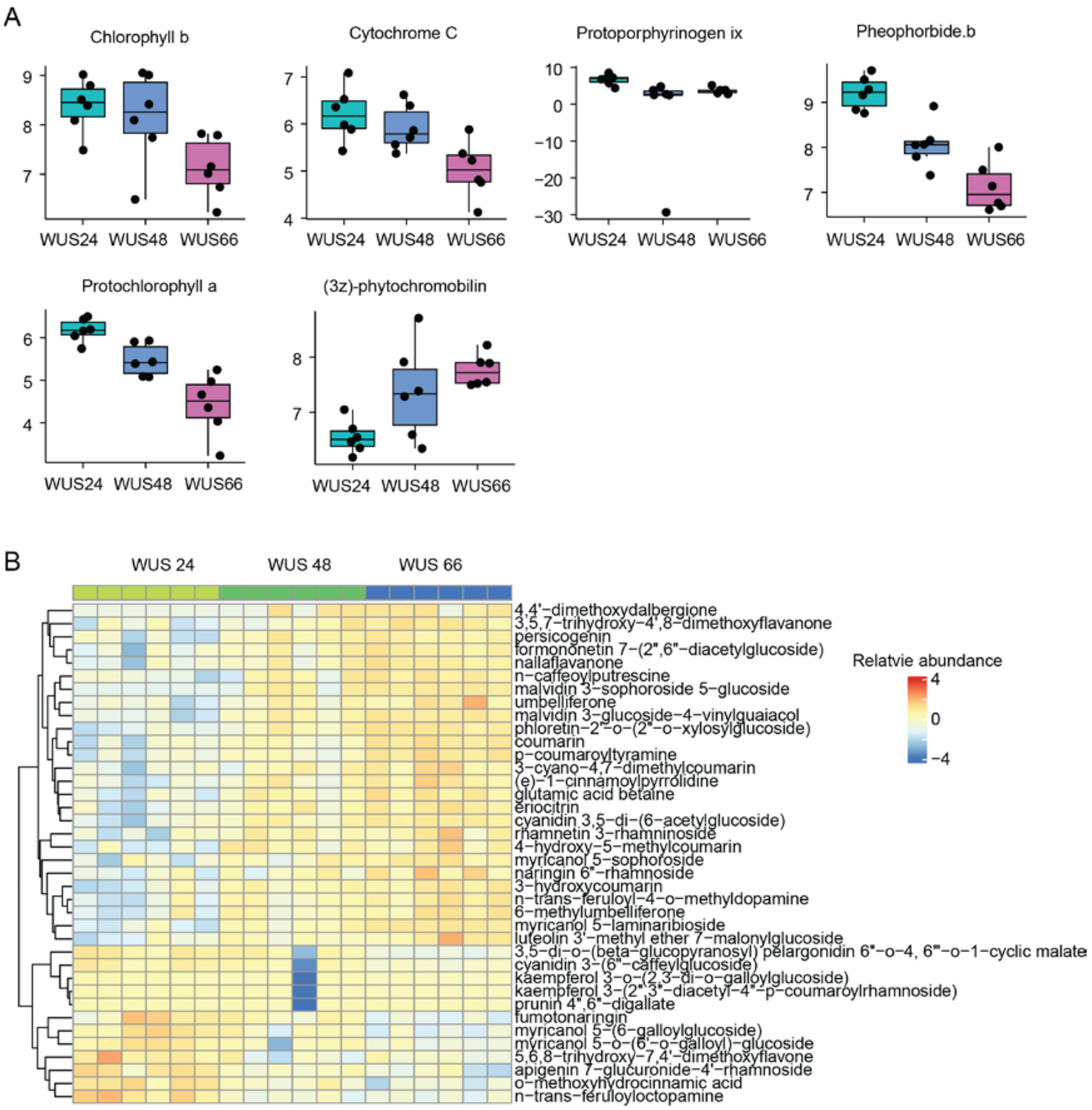

Figure S5. (**A**) Boxplots showed the relative abundance of Chlorophyll b and their derivatives. (**B**) Heatmap overview of the remaining 38 phenylpropanoids. The compounds were scaled across all samples, where blue indicates relatively low expression and red indicates relatively high expression in the respective sample. The compounds underwent unsupervised clustering using complete linkage and Euclidean distance.

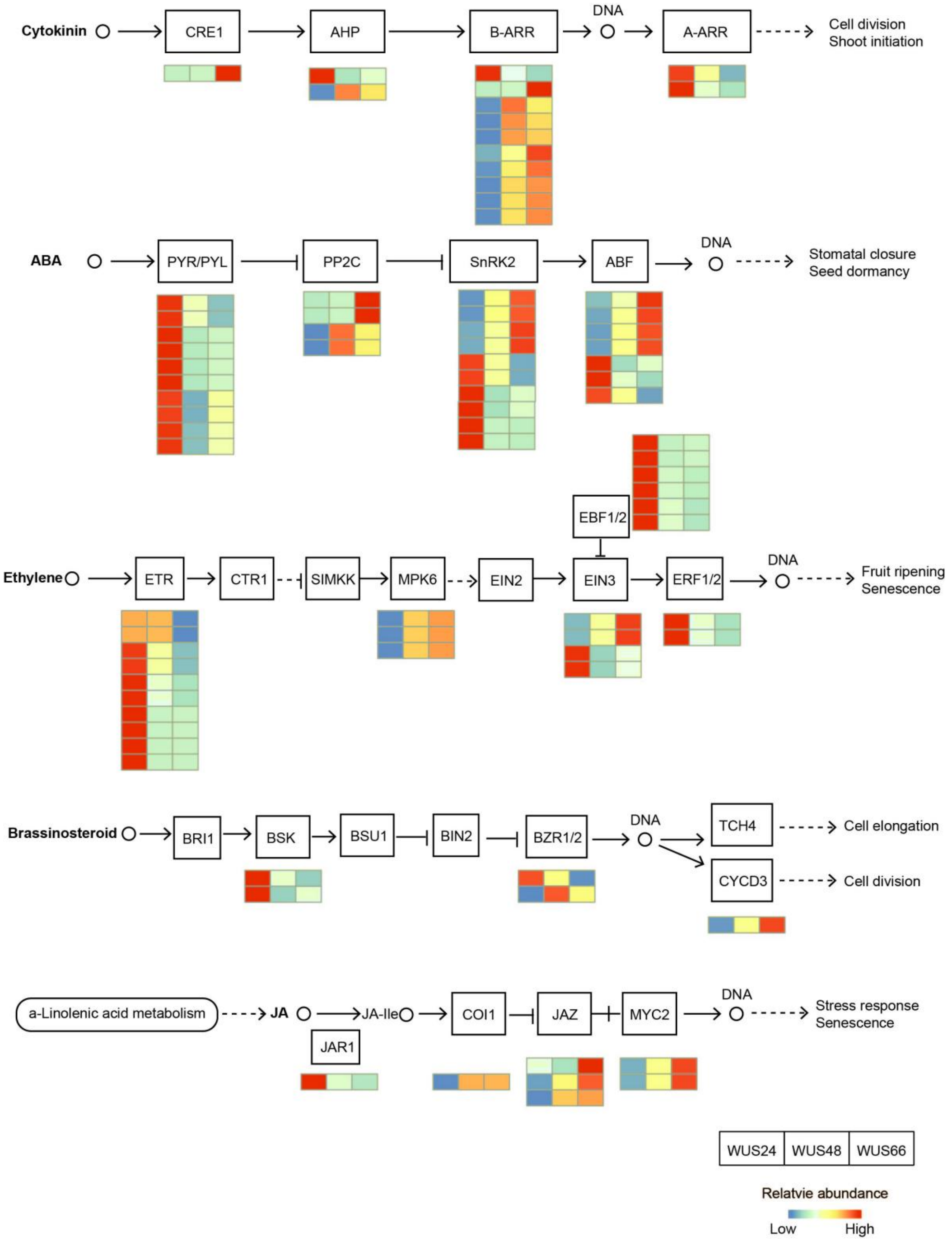

Figure S6. DEGs in plant hormone signaling including cytokinin, ABA, ethylene, brassinosteroid, and jasmonic acid.
